# Key factors influencing multidrug-resistant tuberculosis in patients under anti-tuberculosis treatment in two centres in Burundi: a mixed effect modelling study

**DOI:** 10.1186/s12889-021-12233-2

**Published:** 2021-11-23

**Authors:** Arnaud Iradukunda, Gabin-Pacifique Ndayishimiye, Darlene Sinarinzi, Emmanuel Nene Odjidja, Nestor Ntakaburimvo, Innocent Nshimirimana, Cheilla Izere

**Affiliations:** 1 Department of Medicine, University of Burundi, Bujumbura, PB 1550 Burundi; 2 Department of Statistics, Lake Tanganyika University, Mutanga, PB 5304 Burundi; 3 Royal Society of Tropical Medicine and hygiene, 303-306 High Holborn, London, UK; 4 Department of Medicine, School of Clinical Sciences, Monash University, Wellington Rd, Clayton, VIC 3800 Australia; 5 Department of Computer Mathematics, Clermont Auvergne University, PB 63000 Clermont-Ferrand, France

**Keywords:** Tuberculosis, Multidrug resistant tuberculosis, Bayesian, Bootstrap, Burundi

## Abstract

**Background:**

Despite the World Health Organization efforts to expand access to the tuberculosis treatment, multidrug resistant tuberculosis (MDR-TB) remains a major threat. MDR-TB represents a challenge for clinicians and staff operating in national tuberculosis (TB) programmes/centres. In sub-Saharan African countries including Burundi, MDR-TB coexists with high burden of other communicable and non-communicable diseases, creating a complex public health situation which is difficult to address. Tackling this will require targeted public health intervention based on evidence which well defines the at-risk population. In this study, using data from two referral anti-tuberculosis in Burundi, we model the key factors associated with MDR-TB in Burundi.

**Methods:**

A case-control study was conducted from 1^st^August 2019 to 15^th^ January 2020 in Kibumbu Sanatorium and Bujumbura anti-tuberculosis centres for cases and controls respectively. In all, 180 TB patients were selected, comprising of 60 cases and 120 controls using incidence density selection method. The associated factors were carried out by mixed effect logistic regression. Model performance was assessed by the Area under Curve (AUC). Model was internally validated via bootstrapping with 2000 replications. All analysis were done using R Statistical 3.5.0.

**Results:**

MDR-TB was more identified among patients who lived in rural areas (51.3%), in patients’ residence (69.2%) and among those with a household size of six or more family members (59.5%).

Most of the MDR-TB cases had already been under TB treatment (86.4%), had previous contact with an MDR-TR case (85.0%), consumed tobacco (55.5%) and were diabetic (66.6 %). HIV prevalence was 32.3 % in controls and 67.7 % among cases. After modelling using mixed effects, Residence of patients (aOR= 1.31, 95%C: 1.12-1.80), living in houses with more than 6 family members (aOR= 4.15, 95% C: 3.06-5.39), previous close contact with MDR-TB (aOR= 6.03, 95% C: 4.01-8.12), history of TB treatment (aOR= 2.16, 95% C: 1.06-3.42), tobacco consumption (aOR = 3.17 ,95% C: 2.06-5.45) and underlying diabetes’ ( aOR= 4.09,95% CI = 2.01-16.79) were significantly associated with MDR-TB. With 2000 stratified bootstrap replicates, the model had an excellent predictive performance, accurately predicting 88.15% (95% C: 82.06%-92.8%) of all observations. The coexistence of risk factors to the same patients increases the risk of MDR-TB occurrence. TB patients with no any risk factors had 17.6% of risk to become MDR-TB. That probability was respectively three times and five times higher among diabetic and close contact MDR-TB patients.

**Conclusion:**

The relatively high TB’s prevalence and MDR-TB occurrence in Burundi raises a cause for concern especially in this context where there exist an equally high burden of chronic diseases including malnutrition. Targeting interventions based on these identified risk factors will allow judicious channel of resources and effective public health planning.

**Supplementary Information:**

The online version contains supplementary material available at 10.1186/s12889-021-12233-2.

## Background

Tuberculosis (TB) is one of major causes of death in the world [1]. In 2019, the World Health Organisation (WHO) estimated 10 million new TB cases with an estimated 15% mortality [2, 3]. Generally, it is estimated that 25% of the world’s population are predicted to be infected with TB bacteria and to develop the disease [2, 3]. Most of new tuberculosis cases and death are reported in Southeast Asia and Africa with 44% and 24% respectively [3].

Despite the WHO efforts to expand access to the TB treatments, multi-drug resistant tuberculosis (MDR-TB) which is TB resistant to at least isoniazid and rifampicin [4, 5], remains a major public health threat [6–9]. Worldwide, 484,000 of MDR cases were reported in 2018, contributing to 44.21% of TB related deaths. In 2019, 465,000 cases of MDR-TB were reported, representing a 3.9% increase from 2018 [3, 10]. Among these cases, more than a half (62%) were not treated [9].

In Sub-Saharan Africa regions, the MDR-TB’s prevalence among new cases ranges between 0.4% in Tanzania and 4.4% in Uganda [11, 12]. Then, among recurrent cases from these two countries, the MDR-TB prevalence was 3.9% 17.7% respectively [12]. In 2019, 23 persons per every 100,000 in the South Africa population were reported to have MDR-TB [13, 14]. Regarding risk factors, recent studies conducted in Tanzania and Niger showed TB relapse , history of irregular treatment, cigarette smoking, alcohol abuse, treatment failure, underweight and HIV as leads factors of MDR-TB occurrence [15, 16]. Beside the HIV/AIDS burden, the HIV and TB co-infection burden in Sub-Saharan Africa remains also relatively high [17]. To meet the 2020-2035 global targets on TB and MDR-TB elimination, intensified efforts to improve TB diagnosis, TB treatment and TB prevention are required [18].

In Burundi, the Rifampicin resistant tuberculosis (RR-TB) treatment started in 201 3[19]. Nine month short-treatment regimen improved outcomes of RR-TB treatment with only 8.2% of treatment failure [19]. Despite evident progress,recent survey have shown that only half of cases are detected, DR-TB form are spreading [20]

The respective incidences of TB and RR-TB are about 111 and 3.3 per 100 000 population [20, 21]. In 2018, 2600 patients died from TB [19, 21]. Currently, there is no isoniazid resistance test in Burundi and RR-TB patients are treated as MDR-TB patients. Although we know something about MDR-TB, we do not know who are at risk and where they are. Therefore, this study aims to fill this gap via providing this information to support public health planning and improve clinical outcomes for patients.

Specifically, this study aims to identify key factors influencing MDR-TB among tuberculosis patients. It also aims to predict probability of MDR-TB occurrence among DS-TB patients knowing risk factors. This study could provide evidence for planning of the targeted programmatic interventions to reduce burden of multidrug-resistant tuberculosis in Burundi.

## Methods

### Study Design

**Figure 1:**
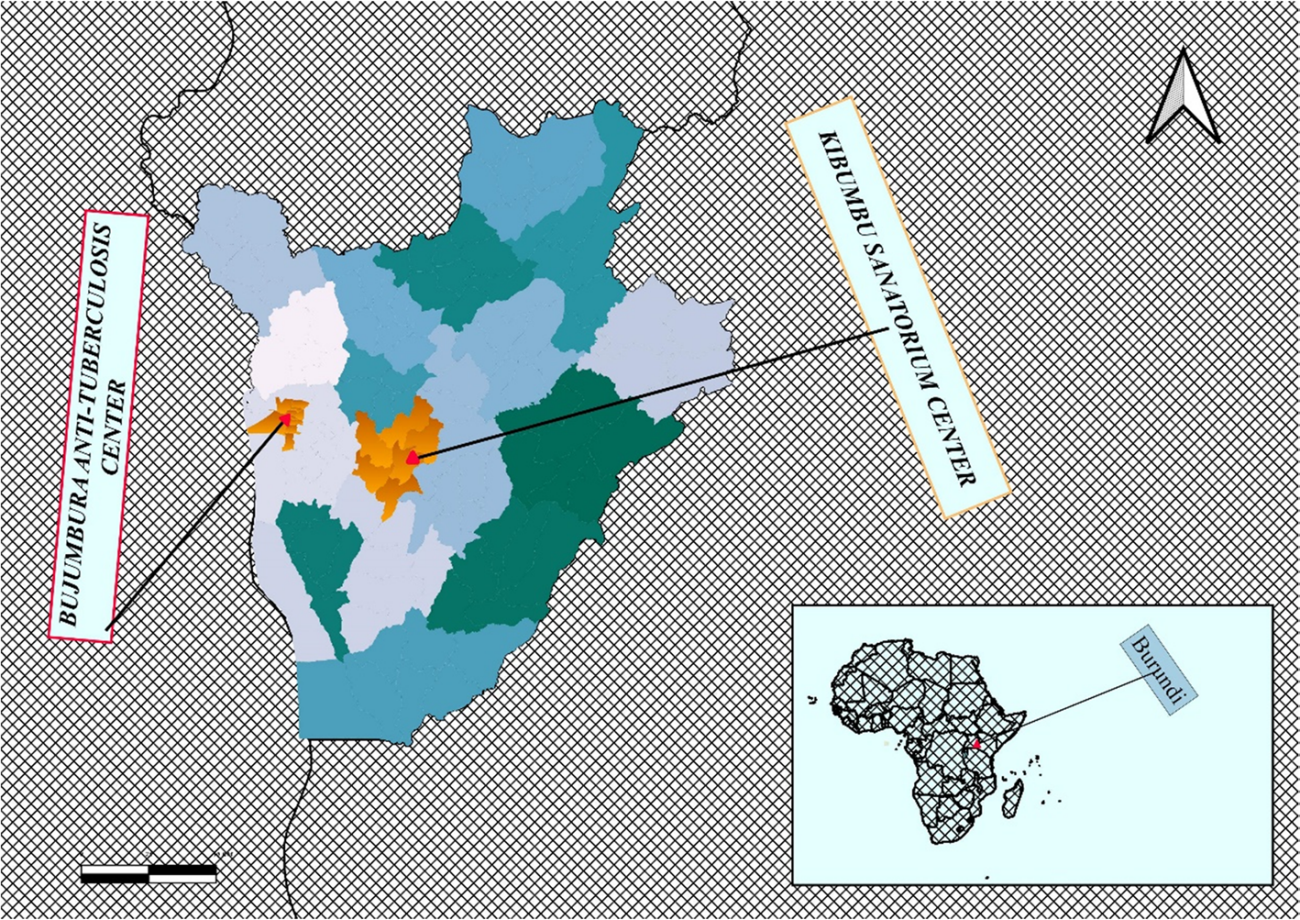
Study area

We conducted a Case-Control study among patients from two Burundian health facilities .The study was conducted from 1^st^August 2019 to 15^th^ January 2020 at the Kibumbu Sanatorium and Bujumbura anti-tuberculosis centre for cases and controls respectively. In Burundi, all TB-patients are treated and monitored by the national TB-program. There are 308 TB-treatment centres, 170 of which provide smear microscopy. The national reference laboratory performed culture and DS-TB is Kimbumbu Sanatorium. Then, RR-TB and MDR-TB patients are referred to Kibumbu which is the only national reference centre for MDR-TB treatment [19]. These centres were chosen in our study because they were the only tertiary reference centres at national level for DS-TB and MDR-TB management in Burundi. Another reason is that one centre was located in urban area and the other in a rural area. A total of 180 TB patients (60 cases and 120 Controls) were selected. The figure below is indicates of the study area (Figure 1).

### Population of study

A sample of confirmed TB patients consisted of the sampling frame. Cases were all patients referred from Bujumbura anti-tuberculosis centre to Kibumbu for MDR-TB treatments during the study period. They were defined by a confirmed pulmonary tuberculosis, a positive bacteriologic and positive gen-Xpert MTB/RIF’s tests with previous DS-TB treatment at Bujumbura anti-tuberculosis centre.

For cases , we selected any patient referred from Bujumbura anti-tuberculosis centre to the national centre for MDR-TB’s the management (Kibumbu Sanatorium) during the period of our study, any patient aged 15 years and older and any patient freely adhering to our study after explanation of the objectives pursued. Then, we excluded from the study cases: any patient admitted before our study period, any patients from other health facility than Bujumbura anti tuberculosis centre, any patient under 15 years old or patient who refused to join our study. As isoniazid resistance test is not available in Burundi, all rifampicin-resistant patients are treated with MDR-TB drugs.

Controls were DS-TB patients on the first line tuberculosis treatment. They were defined by a confirmed pulmonary tuberculosis with a positive bacteriologic and negative gen-Xpert MTB/RIF, no Rifampicin resistance. Controls were at risk of becoming cases and were nearly similar to the cases except for the presence of MDR. Controls were selected using Incidence Density Selection (IDS) method. For controls, we included any patient diagnosed with DS-TB, treated and followed up at Bujumbura antituberculosis during the period of our study, and patient willing to be interviewed. Among them, we excluded any patient diagnosed outside the study period and disagree to be interviewed.

### Sampling methods

The minimal sample size was calculated as detailed in the supplementary document (Supplementary document 1).

Therefore, the cases’ minimal sample size was 50 MDR-TB patients. However, 67 cases of MDR-TB occurred during the period of our study. All of these patients were supposed to be under adequate treatment but not all of them went to Kibumbu for adequate treatment. One of them had comorbidities which required him an intensive care in specialized facility at Bujumbura, four others were lost to follow-up and the two last refused to participate in the study. In total, 180 patients including 60 MDR -TB and 120 DS-TB patients were included in the study .The one case was matched with a control.

### Data collection

Data were collected using forms. We collected different cases’ and controls’ characteristics as summarized in Table 1. For each patient, we collected socio-demographic information including sex, age, marital status, education level, residence; Living conditions and dietary habits information including collective residence, work, number people by house, rooms by house, tobacco consumption, alcohol consumption ; Clinical information including HIV and diabetes status, multidrug resistant tuberculosis close contact and finally therapeutic information including tuberculosis treatment history.

**Table 1:** Sociodemographic and clinical characteristics of the patients

Characteristics	Modalities	Total	Cases	Controls	MDR-TB Proportion
Sex	Women	57	19	38	33.3
	Men	123	41	82	33.3
Age	<45 years	133	45	88	33.8
	≥45 years	47	15	32	31.9
Education level	None	34	5	29	14.7
	Primary	89	34	55	38.2
	Secondary	45	18	27	40.0
	University	12	3	9	25.0
Marital status	Single	63	17	46	26.9
	Married	96	37	59	38.5
	Separated	21	6	14	28.6
Residence	Rural	39	20	19	51.3
	Urban	141	40	101	28.4
Collective résidence	No	167	51	116	30.5
	Yes	13	9	4	69.2
Jobs	Yes	148	55	93	37.2
	No	32	5	27	15.6
People by house	≤6	143	38	105	26.6
	>6	37	22	15	59.5
Room by house	One	32	10	22	31.3
	Two	88	21	67	23.9
	Three	51	25	26	49.0
	Four	7	2	5	28.6
	Five	2	2	0	100
People by room	One	10	7	3	70.0
	Two	87	23	64	26.4
	Three	67	24	43	35.8
	Four	13	5	8	38.5
	Five	3	1	2	33.3
HIV	No	149	50	99	33.5
	Yes	31	10	21	32.3
Diabetes	No	174	56	118	32.2
	Yes	6	4	2	66.6
TB treatment history	No	158	41	107	25.9
	Yes	22	19	13	86.4
MDR-TB close contact	No	160	43	117	26.9
	Yes	20	17	3	85.0
Tabacco	No	143	38	105	26.6
	Yes	37	22	15	55.5
Alcohol	No	54	12	42	22.2
	Yes	126	48	78	38.1
*Total*		**180**	**60**	**120**	**33.3**

### Statistical analysis

Data analysis were done in different steps. In the first step, a descriptive analysis was done. Patients’ characteristics were presented in a summarized table. In the second step, we evaluated the factors associated with MDR-TB using generalized linear model: binary logistic regression with mixed effects. Factors with *p*-value less than 25% and potential cofounding variables were included in multivariate logistic regression. The adjusted odds and confidence intervals were calculated .A *p*-value less than 5% was considered statistically significant [13, 22]. Last step, we calculated the predictive power of the final model and probabilities of MDR-TB occurrence. The risk estimate equation for multivariate logistic regression is detailed below: (Supplementary document 2).

The Wald test was used to determine significance of independent variables on the outcome [23]. To select the best model for this study, the Bayesian Information Criterion (BIC) based on adjustment [24–26] .The BIC’s parameters are detailed in the supplementary document (Supplementary document 3).

The best model was the one with lowest BIC’s value. The relevance of the final model to make prediction was assessed using the Pearson residuals test. Receiving Operating characteristics (ROC) and Area under Curve (AUC) were respectively used to evaluate performance and predictive power of the model. Furthermore, the ROC was used to determine the discriminatory performance of the model, determining the false positive and false negative rates. The Mann Whitney statistics showed that the two distributions were offset: Drug susceptible patients had an average of high scores of classification than MDR-TB patients. Each individual’s score was ranked in ascending order. Thus, the AUC which determined the number of observations accurately predicted was calculated. Multicollinearity between the independent variables was assessed using the Generalised Variance Inflation Factor (GVIF) which shows how the variance of an estimated parameter increases when the independent variables are correlated. One way of making these GVIFs comparable across their dimensions, we calculated the standardised GVIF as following: (Supplementary document 4).

R statistical software (3.5.0) was used in processing and data analysis, lme4, and forest model packages were also for further analysis [27]. In validating internal performance of the model, a bootstrapping procedure with 2000 replications was conducted. The above statistical analysis was selected due to the nature of the observations data. As unmatched case control study, we analysed data using unconditional logistic regression [28]. Furthermore, a bootstrapping method was selected as a good way to internally validate the performance of the model, a practice which is considered as good practice.

## Results

### Sociodemographic and clinical characteristics of the patients

This study identified 67 MDR-TB patients who became MDR-TB during the period of our study, of which 89.6% (*n*=60) were included in the study. Among the 180 TB patients, 66. 6% of them were under DS-TB treatment (Controls). The median and mean age of patients were 33.00 and 36.25 years respectively. The youngest patient was 15 year old and maximum age was 85 years. Half of women and men were respectively under 29 and 35 years. A third of the cases and controls were below age 45.

The table below shows characteristics of patients according to their drug susceptibility and multi resistance profile (Table 1) More than 60 % of both cases (68.3 %) and controls (68.3%) were men (Table 1). High MDR-TB’s prevalence, more than 50%, was observed in patients who live in rural zone, in collective residence, in household with more than six people by house, patients with TB treatment history and in diabetic patients (Table 1). The table below shows determinants factors of MDR-TB the multivariate regression.

**Figure 2:**
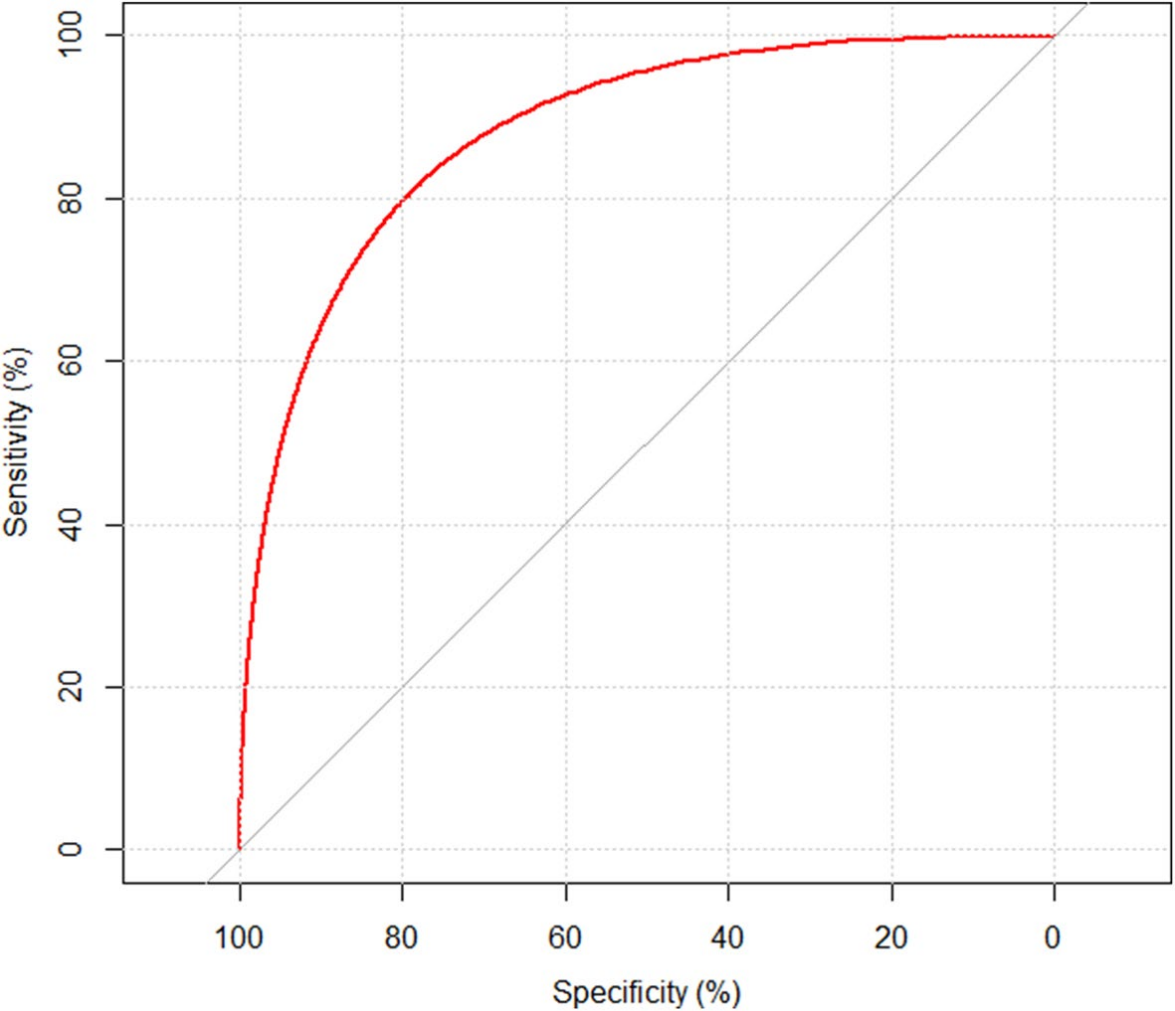
Area under curve

After controlling the cofounding factors using fix and random effects, adjusted odds ratio, their confidence intervals (CI) with a corresponding *p*-value were derived (Table 2). The adjusted model showed six factors which are significantly associated with MDR-TB: Patient’s residence (aOR=1.31; 95% C:1.12-1.80; *p*=0.020), people per house (OR=4.15; 9 5% C:3.06-5. 39; *p*<0.001), MDR-TB close contact (aOR=6.03; 95% C:4.01-8.12;*p*<0.001), T B treatment history (aOR=2.16; 95% C:1.06-3.42;*p*<0.001), Tobacco (aOR=3.17; 95% CI: 2.06-5.45; *p*<0.001) and Diabetes (aOR=4.09; 95% C:2.01-6.79;*p*=0,030). However some socio -demographic factors such as Sex, marital status and Age of our TB patient were not significantly associated with MDR-TB. Furthermore, the number of rooms by house, a collective residence and patient’s work are not significantly associated with MDR-TB. The Figs 1 and 2 below shows reapectivly the Area Under Curve and Outliers of our prediction model .

**Table 2:** Logistic regressions

Variables	Modalities	Total	aOR	95% CI	***p***-value
Residence	Urban	141	Reference		
	Rural	39	1.31	[1.12-1.80]	0.020
People per house	≤6	143	Reference		
	>6	37	4.15	[3.06-5.39]	<0.001
MDR-TB close contact	No	160	Reference		
	Yes	20	6.03	[4.01-8.12]	<0.001
History TB treatment	No	148	Reference		
	Yes	32	2.16	[1.06-3.42]	<0.001
Tobacco	No	143	Reference		
	Yes	37	3.17	[2.06-5.45]	<0.001
Diabetes	No	174	Reference		
	Yes	6	4.09	[2.01-6.79]	0.030

*AOR* Adjusted Odds ratio, *CI* Confidence interval, *p p* value

**Figure 3:**
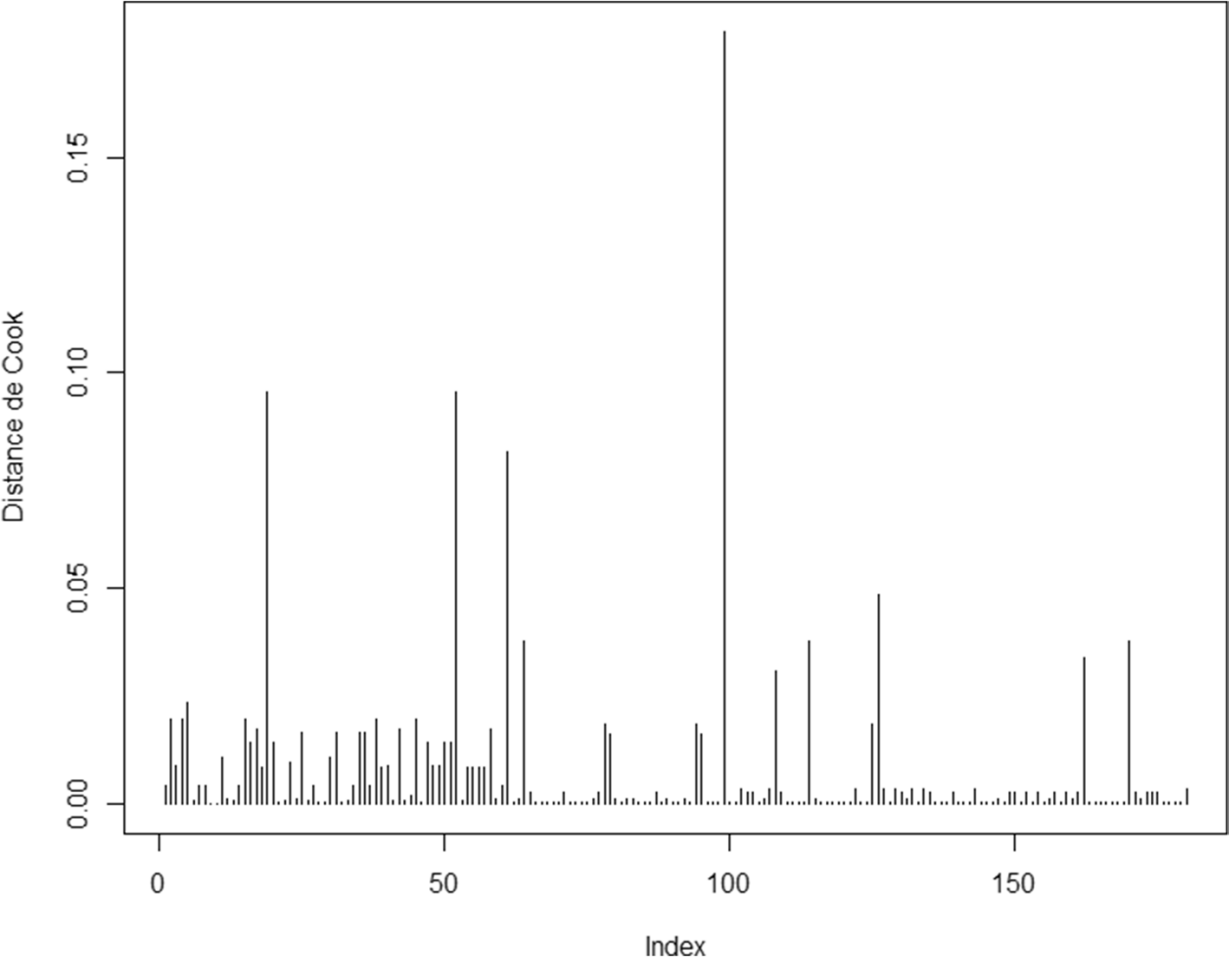
Cook’s distance

The model’s predictive power evaluated by 2000 stratified bootstrap replicates and binormal smoothing shows that the AUC was 88.15 % (95% C, 82.06%-92.8%). It means that the model has an excellent discrimination (Figure 2) .Cook’s distance shows that the influential points are not numerous: only 3 points (19, 52 and 99) are outliers (Figure 3).

Pearson residuals test of (X^2^= 183.86, df =173) was determined with a *p*-value of 0.272 which shows the model was well adjusted on the observations. A McFadden’s statistic (R^2^: 0.346) also indicates that this model has a good fit. The table 3 below shows collinearity assessment among predictors (Table 3)

**Table 3:** Generalized Variance Inflation Factor

Variables	GVIF	ddl	GVIFS
Residence	1,04	1	1,09
People per house	1,50	1	1,11
MDR-TB close contact	1,12	1	1,06
History of TB treatment	1,46	1	1,09
Tobacco	1,21	1	1,06
Diabetes	1,05	1	1,11

According to results of the Table 3, no GVIFS value exceed 3, hence there was no multi collinearity among the independent variables.

Using K-folds method (K=6), the cross-validation error rate each group were 0.33, 0.33, 0.13, 0.20, 0.23 and 0.33 respectively. The mean error, which is the best error rate estimator than the substitution error rate, was 0.2611.The Table 4 below shows predicted propabilities of MDR-TB occurrence knowing risk factors.

**Table 4:** Predictions

Ind.	Residence	PH	MTCC	THT	Tobacco	Diabetes	*P* value
1*	Urban	<6	No	No	No	No	0.176
2	Urban	<6	No	No	No	Yes	0.657
3	Urban	<6	No	Yes	No	No	0.888
4	Rural	<6	Yes	No	No	No	0.998
5	Rural	<6	Yes	No	No	No	0.999
6	Rural	>6	Yes	Yes	Yes	Yes	≈0.100

*PH* People per house, *MTCC* MDR-TB close contact, THT TB history treatment

TB patients with no any risk factors had 17.6% of risk to become MDR-TB. That probability was three times higher among diabetic patients (Table 4). Also, that probability were five times higher in patients with MDR TB close contact. The coexistence of risk factors to the same patients increases the risk of MDR-TB occurrence.

## Discussion

This study aims to identify and assess the various risk factors associated with MDR-TB in the context of Burundi. First, we determined the proportions of MDR-TB in different categories of patients. Secondly we identified main factors associated with MDR-TB. Lastly, we predicted the probabilities of MDR-TB occurrence knowing risk factors.

In this study, patient’s residence, high number of people by house, MDR-TB close contact, TB history treatment, Tobacco consumption and underlying of Diabetes were identified main risk factors of MDR-TB. These result are consistent with previous studies [29, 30]. Specifically, this study showed that patients who had close contact with known MDR-TB patients were about 6.03 times more likely to develop MDR-TB than control patients. Similar findings have been reported in previous studies elsewhere in Africa (Ethiopia) and South America (Peru) [31, 32]. Others findings which are in line with our findings have been reported in three recent studies conducted in Ethiopia , Sudan and Namibia which showed that close contact with known MDR-TB patients was strongly associated with MDR-TB [33–35]. In low-income countries, these findings can be explained by delays in diagnosis and treatment of MDR-TB patients. Therefore, health education for TB patients and their family members on TB infection is necessary to control the MDR-TB spread in among household members.

Patients with history of TB treatment had about 2 times higher the risk to become MDR-TB. This finding was consistent with studies conducted in Central Nepal, Brazil and South Africa [33–35]. MDR could be due to the non-compliance with previous TB treatment coupled with low education level affects indirectly the well use of TB treatment [36]. More than 85% of retreated TB patients were MDR-TB in our study. Those results are comparable to those reported in a previous study conducted in China which showed a high prevalence of multidrug resistance among retreated TB [34]. As evidenced in this study many previous studies conducted in Ethiopia and Worldwide by the WHO showed that MDR-TB was more identified in patients with TB treatment history [37–42]. These findings can be explained by sub-optimal availability of diagnostic and treatment coupled with poor infection prevention measures at health care facility level and at home.

Contrary to this study, findings of other studies reported that HIV was as principal risk factor. The TB-HIV co-infection found in this study is comparable with the TB –HIV’s prevalence reported in previous studies conducted in different parts of Ethiopia [32, 43] . This study shows that HIV-positive patients are less among MDR-TB than drug susceptible TB patients. These findings are also in line with results reported in a study conducted in India [44]. Developing MDR-TB depends on type of TB patients during their previous treatment [45]. Low prevalence of TB-HIV co-infection can be explained by the high mortality rate among MDR-TB. However, this study did not find any association of MDR-TB and age, sex, education level, marital or employment status. Number of people living in the same room and HIV infection were significantly associated with development of MDR-TB .Patient with no risk factors had 17.6% of risk to become MDR-TB, this probability was three times higher in diabetic patients. Also, this probabilities is 5 times higher in patients with MDR-TB close contact with MDR TB patients. The coexistence of risk factors to the same patients increase the risk of becoming MDR-TB patients

The major strength of this study was inclusion of patients from two national areas, and selecting Cases and Controls based on the result of biological test and the use of strong statistical method: Incidence Density Selection method. Some increasing of the case control study was achieved by increasing the number of controls (two controls were used). Another strength is combining descriptive and inferential statistics, logistic regressions with mixt effects, Wald test and Pearson's Chi-squared test with Yates' continuity correction, Cross validation and decision tree. The absence of isoniazid resistance test in the country and treatment of all RR-TB patients as MDR-TB patients are the main limits of this study. Others limitations of this study should be noted when using findings of this study to inform policy. First, the study may have suffered from a sample size due to the nature of conditions under study. To mitigate the possible impact of this, a bootstrapping technique was adopted to replicate dataset by several observations to confirm results. Another important limitation worth noting is the possibility of a recall bias as responses on background characteristics was solicited via recall. In addition, some information on uses of different substances were not sufficient. A Bayesian regression based on Markov chain Hamiltonian Monte Carlo simulates and Langevin algorithms could give precision in the estimation of model’s parameters and Bayesian credibility intervals as such these methods are recommended for future research.

## Conclusion

The current study shows factors influencing the occurrence MDR-TB among patients. They include living conditions, Sociodemographic and clinical factors. .MDR-TB is more observed in patients who live in rural zone, in collective residence, in more than six people per house, one people by room, people with TB treatment history and in people with diabetes. Other factors of MDR-TB occurrence were: patient’s residence, high household size, MDR-TB close contact, TB history treatment, Tobacco consumption and Diabetes was identified as strongest association with MDR-TB, which are consistent with previous studies.

In Burundi resources are scarce. Tackling the high burden of tuberculosis and MDR-TB should be based on instituting systems for early detection and treatment. At the community level, efforts should be channelled towards intensifying innovative and inclusive health promotion aimed at behavioural change in people lifestyle and living conditions. At the health system, identification of factors could allow those at high risks to be identified early and well-targeted with the needed treatment with good health care services. Finally, not only adequate treatment, prevention based on risk factors and the overall health systems strengthening will reduce the number of TB infections and Multidrug resistance. This will ensure sustainability and effectiveness of public health interventions aimed at tackling TB and MDR-TB along with other high burden infectious and chronic diseases.

## Supplementary Information

**Additional file 1.**

**Additional file 2.**

**Additional file 3.**

**Additional file 4.**

## Data Availability

The dataset and materials used in this study are available from the corresponding author on reasonable request

## Abbreviations

AIDS: Acquired Immunodeficiency Syndrome
aOR: Adjusted Odds Ratio
AUC: Area Under Curve
BIC: Bayesian Information Criterion
DS: Drug susceptible
GVIFs: Generalized Variance Inflation Factor
IDS: Incidence Density Selection
Mbt: Mycobacterium tuberculosis
MTCC: Multidrug resistance close contact
MDR: Multidrug resistant
HIV: Human Immunodeficiency Virus
INH: Izoniasid
PZA: Pyrazinamid
RIF: Rifampicin
MDR: Multidrug resistant
WHO: World Health Organisation
PH: People per house
ROC: Receiving Operating Characteristics
Rce: Residence
RR: Resistance to rifampicin
TB: Tuberculosis
THT: Tuberculosis history treatment
(c): Candidate
TB: Tuberculosis
