# Supplementary material for: Key factors influencing multidrug-resistant tuberculosis in patients under anti-tuberculosis treatment in two centres in Burundi: a mixed effect modelling study
Source: BMC Public Health. 2021 Nov 23;21:2142. doi: 10.1186/s12889-021-12233-2 (PMC8609742; doi:10.1186/s12889-021-12233-2)
Supplement: Supplementary file 1 — Additional file 1. [file 12889_2021_12233_MOESM1_ESM.docx]

**Sample Size Calculation**

$\mathrm{n}=\frac{\mathrm{Z}_{1-\frac{\mathrm{\alpha}}{2}}^{2}\times\mathrm{p}\left(1-\mathrm{p}\right)\times\kappa}{\mathrm{d}^{2}} \left(1\right)$ $\mathrm{Z}_{1-\frac{\mathrm{\alpha}}{2}}$ The normal distribution quintile with 95% of confidence (1.96), $n$minimal size sample,$p$ prevalence of MDR-TB, $\kappa$: No response rate (10%) and $\mathrm{d}$ acceptable margin (5%). As the prevalence of MDR-TB is unknown in Burundi, a WHO’s estimated value of $p=0.03$ was used.
