# Supplementary material for: Key factors influencing multidrug-resistant tuberculosis in patients under anti-tuberculosis treatment in two centres in Burundi: a mixed effect modelling study
Source: BMC Public Health. 2021 Nov 23;21:2142. doi: 10.1186/s12889-021-12233-2 (PMC8609742; doi:10.1186/s12889-021-12233-2)
Supplement: Supplementary file 2 — Additional file 2. [file 12889_2021_12233_MOESM2_ESM.docx]

**Risk calculation for multiple logistic regression**

$$\log\left[\frac{\mathrm{p}}{1-\mathrm{p}}\right]=\mathrm{\beta}_{0}+\mathrm{\beta}_{1}\mathrm{X}_{1}+.. .+\mathrm{\beta}_{\mathrm{k}}\mathrm{X}_{\mathrm{k}}+\mathrm{\varepsilon} \left(3\right)$$

Where $p$is outcome realization probability, $\beta_{0}$intercept, $\beta_{i}$coefficients, $X_{i}$independents variables and $\text{ε}$error. All significant variables on 15% threshold in univariates analysis were introduced in multivariate logistic modelling to determine a combined effect on the outcome. Finally, the predictor variables of the model were manually selected step by step using backwards elimination method on a 5% threshold. The model include $\beta_{0}$captures fixed effects and $\beta_{i}$ which detects random effects on probabilities of multidrug tuberculosis development.
