# Supplementary material for: Key factors influencing multidrug-resistant tuberculosis in patients under anti-tuberculosis treatment in two centres in Burundi: a mixed effect modelling study
Source: BMC Public Health. 2021 Nov 23;21:2142. doi: 10.1186/s12889-021-12233-2 (PMC8609742; doi:10.1186/s12889-021-12233-2)
Supplement: Supplementary file 3 — Additional file 3. [file 12889_2021_12233_MOESM3_ESM.docx]

**Bayesian Information Criterion (BIC) equation**

$\mathrm{BIC}=\mathrm{p}\times\ln(\mathrm{n}) -2\ln\left(\mathrm{L}\right) \left(4\right)$Where $\text{p}$model parameters number, $\mathrm{n}$ observations number, and {\displaystyle {\mathit {AIC}}=2k-2\ln(L)}$\text{L}$refers to the maximum value of the likelihood function of the model.
