# Supplementary material for: Key factors influencing multidrug-resistant tuberculosis in patients under anti-tuberculosis treatment in two centres in Burundi: a mixed effect modelling study
Source: BMC Public Health. 2021 Nov 23;21:2142. doi: 10.1186/s12889-021-12233-2 (PMC8609742; doi:10.1186/s12889-021-12233-2)
Supplement: Supplementary file 4 — Additional file 4. [file 12889_2021_12233_MOESM4_ESM.docx]

**Generalised Variance Inflation Factor (GVIF) Equation**

$\mathrm{GVIFS}=\mathrm{GVIF}^{\frac{1}{2\times\mathrm{ddl}} } \left(5\right)$ $\mathrm{ddl}$Being the number of degrees of freedom corresponding to the GVIF. A value greater than 3 (or 10 at the limit) for this indicator shows the presence of multicolinearity in the data.
